# An Unexpected Outcome of Streptococcus sanguinis Endocarditis Associated With Orthodontic Bracing in a Young Healthy Patient

**DOI:** 10.7759/cureus.39864

**Published:** 2023-06-02

**Authors:** Ali Rahman, Sura Alqaisi, Jayant Nath

**Affiliations:** 1 Internal Medicine, Mather Hospital, Northwell Health, Port Jefferson, USA; 2 Internal Medicine, Memorial Healthcare, Pembroke Pines, USA; 3 Cardiology/Imaging, Memorial Healthcare, Pembroke Pines, USA

**Keywords:** surgical replacement of valve, valvular vegetation, infective endocarditis, streptococcus sanguinis, orthodontics

## Abstract

We present a case of *Streptococcus sanguinis* endocarditis in a 26-year-old female following orthodontic bracing. The rarity and debilitating consequences of endocarditis caused by *Streptococcus sanguinis* are elaborated. The patient exhibited severe regurgitation with the eccentric posteriorly directed flow, leading to significant cardiac strain, further accentuated by systolic flow reversal in the right superior pulmonary vein. Surgical intervention, including mitral valve replacement, was crucial in addressing the underlying infection, restoring valve function, and preventing further complications. However, a second mitral valve replacement was performed due to recurrent bioprosthesis endocarditis. This case underscores the unique challenges of *Streptococcus sanguinis* endocarditis, emphasizing the need for a multidisciplinary approach and individualized decision-making to optimize patient care.

## Introduction

Orthodontic treatment is a standard dental procedure that involves the use of braces to correct dental malocclusions. Although the procedure is generally safe, there have been reported cases of orthodontic treatment leading to infections and systemic complications, such as bacteremia and infective endocarditis (IE). Orthodontic treatment involves using wires, brackets, and bands to move teeth into the desired position. These appliances create an environment where bacteria can grow and accumulate, increasing the risk of bacteremia. The risk of bacteremia is further increased during the adjustment phase of orthodontic treatment when the wires and brackets are tightened. The bacteria can enter the bloodstream through little gums or oral mucosa wounds, leading to bacteremia [1]. We present a case of a 26-year-old female with a history of asthma who underwent orthodontic bracing and subsequently developed frequent upper respiratory tract infections. Our investigation revealed vegetation on the mitral valve, indicating infective endocarditis.

*Streptococcus sanguinis* is a commensal bacterium found in the oral cavity, which can become pathogenic under certain conditions, such as during orthodontic treatment. This bacterium is one of the most common causes of infective endocarditis, particularly in patients with underlying cardiac conditions or compromised immune systems [2]. The most common bacteria associated with infective endocarditis (IE) following dental procedures are viridans group streptococci, which includes *Streptococcus sanguinis*, *Streptococcus mutans*, and *Streptococcus mitis*. Following dental procedures, other bacteria that can cause IE include *Staphylococcus aureus*, coagulase-negative staphylococci, and enterococci [2,3]. The likelihood of *Streptococcus sanguinis* causing endocarditis depends on several factors, including the patient’s risk factors and the nature of the dental procedure. In general, *S. sanguinis* is considered a relatively low-risk bacteria for causing IE, as it is less likely to cause significant damage to heart valves than other bacteria such as *S. aureus* [2].

Patients with underlying medical conditions, such as cardiac abnormalities or compromised immune systems, are at increased risk of developing infections following orthodontic treatment. In particular, patients with congenital heart disease (CHD), rheumatic heart disease, or prosthetic heart valves are at higher risk of developing infective endocarditis. Patients with weakened immune systems, such as those undergoing chemotherapy or with human immunodeficiency virus (HIV)/acquired immunodeficiency syndrome (AIDS), are also at increased risk of developing infective endocarditis following orthodontic treatment. The American Heart Association (AHA) recommends that patients at increased risk of infective endocarditis receive antibiotic prophylaxis before dental procedures that may cause bacteremia [4]. Additionally, dental professionals should follow strict infection control measures, including proper sterilization of instruments and surfaces, to minimize the risk of infection.

The AHA and the European Society of Cardiology (ESC) have issued guidelines on antibiotic prophylaxis before dental procedures to prevent infective endocarditis in at-risk patients, including those undergoing orthodontic procedures. According to the 2007 AHA guidelines [5], antibiotic prophylaxis should be considered for patients with the following conditions: prosthetic cardiac valve or prosthetic material used for cardiac valve repair; previous infective endocarditis; congenital heart disease (CHD) with unrepaired or incompletely repaired cyanotic CHD, including palliative shunts and conduits; completely repaired congenital heart defect with prosthetic material or device, whether placed by surgery or by catheter intervention, during the first six months after the procedure; repaired CHD with residual defects at the site or adjacent to the area of a prosthetic patch or prosthetic device; and cardiac transplantation with valvulopathy. For patients with these conditions undergoing orthodontic procedures, the AHA recommends antibiotic prophylaxis with amoxicillin or clindamycin, except in cases of a penicillin allergy. The ESC guidelines, last updated in 2015, recommend antibiotic prophylaxis for patients with the following conditions: prosthetic heart valves, including transcatheter valves and homografts; previous infective endocarditis; congenital heart disease, including palliative shunts and conduits; acquired valvular heart disease in the presence of a prosthetic material or device; and cardiac transplantation with valvulopathy. The ESC guidelines recommend antibiotic prophylaxis with amoxicillin or clindamycin for these patients undergoing dental procedures, including orthodontic procedures. It is important to note that both sets of guidelines emphasize the importance of individualized decision-making and the need for proper infection control measures to minimize the risk of infection, as no randomized controlled trials are available.

The incidence of IE following orthodontic procedures is generally low, estimated to be less than one in 100,000 cases. However, the risk is higher in patients with pre-existing cardiac abnormalities, compromised immune systems, or a history of infective endocarditis. Several reported cases of IE following orthodontic procedures have been reported, although they are relatively rare. Most cases involved patients with pre-existing cardiac abnormalities or compromised immune systems. The causative microorganisms vary, but *Streptococcus* species are the most commonly reported. It is important to note that while the incidence of IE following orthodontic procedures is low, the potential consequences of the infection can be severe.

## Case presentation

A 26-year-old female patient with a past medical history of childhood asthma was admitted for cough and headache. One week ago, she presented to the hospital with a fever associated with a clear, productive cough and was discharged home the same day. She reported that she continued to have on-and-off headaches, chills, and a productive persistent cough. She denied chest pain or shortness of breath on room air. She has had frequent recurrent fevers and upper respiratory infections over a period of six months after her orthodontic brace was placed. She, therefore, presented multiple times to urgent care and was prescribed antibiotics, which only mildly helped. On arrival at the emergency department (EMD), the patient was afebrile with a saturation of 98% on room air, heart rate of 100 beats per minute, and blood pressure of 95 over 60 mmHg. Her physical examination was unremarkable; however, a grade IV systolic murmur in the mitral area was noted upon chest auscultation.

Laboratory tests were significant for leukocytosis (Table 1). Two blood cultures were drawn from the patient’s arm using an aseptic technique, one set from each arm, inoculated onto culture media, and incubated at 37°C for seven days. After 48 hours of incubation, the blood cultures showed the growth of small alpha-hemolytic colonies on the agar plate, identified as *Streptococcus sanguinis* by Gram staining and biochemical tests, which was sensitive to ceftriaxone antimicrobial susceptibility testing (AST) (Table 2). An infectious disease specialist saw the patient and recommended starting intravenous ceftriaxone 2 g daily and performing transthoracic echocardiography (TTE).

**Table 1:** Laboratory results WBC, white blood cell; BUN, blood urea nitrogen; ESR, erythrocyte sedimentation rate; SARS-CoV-2, severe acute respiratory syndrome coronavirus 2; PCR, polymerase chain reaction

Laboratory tests	Values	Normal range
WBC (×10^9^/L)	18	4.5-11
Hemoglobin (g/dL)	12	12-16
Platelets (×10^9^/L)	155	130-400
Neutrophils (%)	85	40-60
Lymphocytes (%)	8.2	20-40
Monocytes (%)	5	1.7-9.3
Eosinophils (%)	1	0-5
Basophils (%)	0.8	0-3
Sodium (mmol/L)	142	137-145
Potassium (mmol/L)	4.2	3.5-5.2
Chloride (mmol/L)	99	98-107
Carbon dioxide (mmol/L)	22	22-30
BUN (mg/dL)	11	7-17
Creatinine (mg/dL)	0.9	0.52-1.04
ESR (mm/hour)	400	0-29
D-dimer (ng/mL)	56	<250
Ferritin (ng/mL)	110	12-50
Fibrinogen (mg/dL)	220	200-400
SARS-CoV-2 PCR	Negative	Negative

**Table 2:** Antimicrobial susceptibility test

Antimicrobial agent		*Streptococcus sanguinis* (*Streptococcus viridans* group)
Ceftriaxone	0.190 ug/mL	Sensitive
Clindamycin	0.221 ug/mL	Sensitive
Erythromycin	0.450 ug/mL	Sensitive
Levofloxacin	0.300 ug/mL	Sensitive
Penicillin	0.380 ug/mL	Intermediate
Vancomycin	0.115 ug/ml	Sensitive

**Figure 1:**
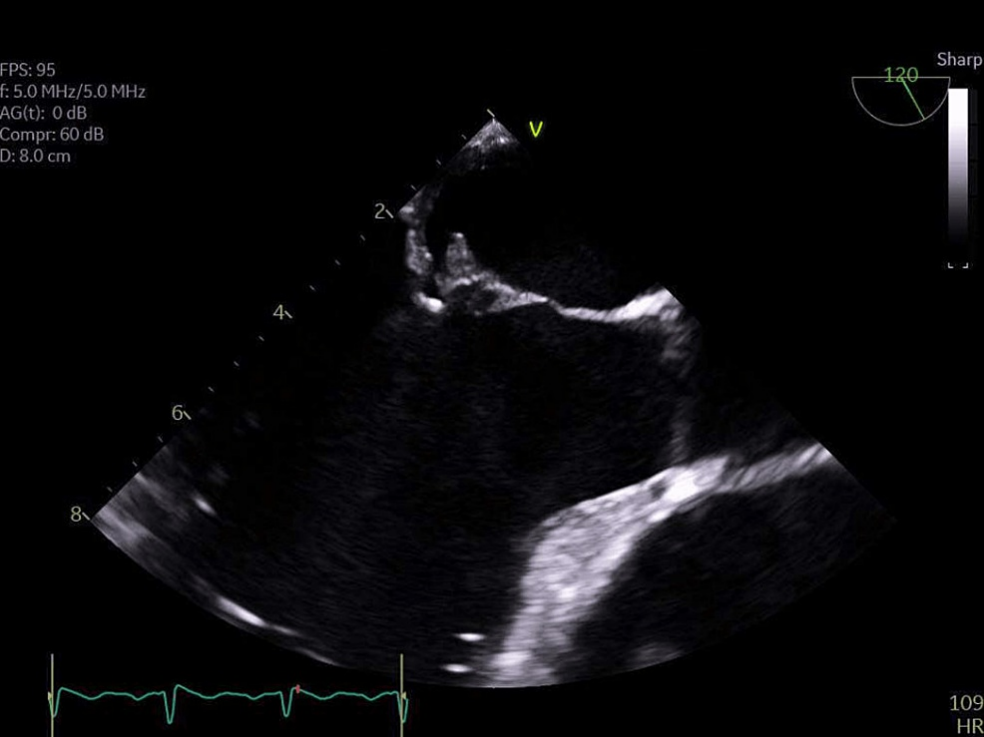
TEE image showing vegetations on the native mitral valve TEE, transesophageal echocardiogram

TTE revealed a left ventricle (LV) ejection fraction of 67%, normal LV diastolic function, severely dilated left atrium (LA), thickened anterior leaflet of the mitral valve with posterior leaflet tethered with restricted motion, moderate to severe mitral valve regurgitation, trivial pericardial effusion, and normal right ventricle (RV) systolic pressure and diastolic function. Then, the patient was evaluated by the cardiology team, who recommended a transesophageal echocardiogram (TEE) to investigate the condition further, confirming the mitral valve infective endocarditis diagnosis. Multiple mobile echo densities were seen on the atrial aspect of both anterior and posterior leaflets of the mitral valve, with the largest measuring 0.8 × 0.4 cm, suggestive of vegetation. No perivalvular abscess was observed. Additionally, part of the anterior leaflet was frail, causing eccentric posteriorly directed severe regurgitation with systolic flow reversal in the right superior pulmonary vein. No other significant valvular regurgitation, stenosis, or vegetation was observed on other valves. There was no significant pericardial effusion (Figure 1).

**Figure 2:**
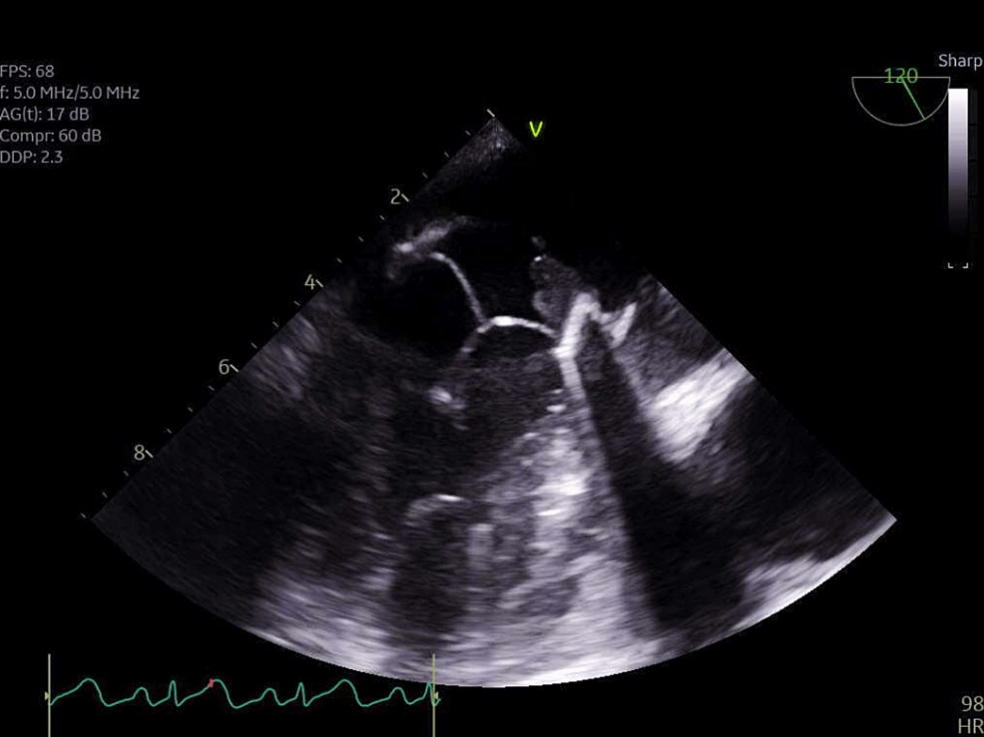
TEE image showing vegetations on the bioprosthetic mitral valve TEE, transesophageal echocardiogram

The patient underwent a surgical procedure to replace the mitral valve with a 31 mm Edwards pericardial mitral valve. The left atrial appendage was also excluded using a 45 mm AtriCure appendage clip. Following the procedure, the patient was found to be febrile. A repeated echocardiogram showed that the left ventricle (LV) had a normal ejection fraction, cavity size, and shape. The right ventricle (RV) had a normal cavity size and normal function. The aortic valve appeared structurally normal, without any stenosis, regurgitation, or vegetation. The mitral valve, previously replaced with a 31 mm Edwards pericardial mitral valve, showed no stenosis but had a trace regurgitation. A small, mobile vegetation measuring 0.5 × 0.8 cm was found on the atrial side of the anterior leaflet. The tricuspid valve appeared normal, and there was no pericardial effusion (Figure 2). Subsequently, the patient underwent another procedure to replace the mitral valve due to recurring bioprosthetic mitral valve endocarditis.

The patient was monitored on the telemetry unit and was evaluated daily by the hospital medicine, infectious disease, and cardiology team. She had multiple repeated blood cultures throughout her hospital stay, which yielded no bacterial growth. The patient was advised to receive a course of intravenous ceftriaxone 2 g daily for six weeks and then will have to follow up in cardiology rehabilitation.

## Discussion

*Streptococcus sanguinis* endocarditis is a relatively rare condition. The presented case of a 26-year-old female diagnosed with this infection following orthodontic bracing adds to the limited literature on this topic. Firstly, the patient’s age, in this case, was noteworthy. Endocarditis is typically more common in older individuals with predisposing factors such as pre-existing heart conditions or intravenous drug use [6]. However, in this case, the patient was only 26 years old, without any underlying cardiac abnormalities. This contrasts with many published studies reporting cases of endocarditis primarily occurring in older age groups, suggesting that *Streptococcus sanguinis* endocarditis in a young individual without predisposing factors is rare but can have debilitating consequences.

Furthermore, the presumed source of infection, in this case, was orthodontic bracing. While dental procedures have been recognized as potential sources of endocarditis, the specific association with *Streptococcus sanguinis* is less commonly reported in the literature [7]. This highlights the case’s uniqueness and emphasizes the importance of considering even routine dental interventions as potential sources of infection.

Based on the Duke criteria, the diagnosis of infective endocarditis, in this case, was confirmed through two major criteria: identifying the vegetation through echocardiography and two positive blood cultures with *S. sanguinis*. Vegetations, characterized by multiple mobile echo densities on the atrial aspect of both the anterior and posterior leaflets, are a hallmark feature of endocarditis [8]. However, the size and location of the vegetation observed in this case, mainly the largest one measuring 0.8 × 0.4 cm, are less frequently reported in the literature. Larger vegetations increase the risk of complications, such as embolization or abscess formation, and may necessitate more aggressive management strategies. Furthermore, echocardiographic findings of severe regurgitation and systolic flow reversal indicate advanced valvular dysfunction and highlight the significant burden that *Streptococcus sanguinis* endocarditis can impose on the heart. The consequences of such severe regurgitation can result in progressive cardiac remodeling, impaired ventricular function, and heart failure if left untreated. Thus, it becomes imperative to address the underlying infection and mitigate the regurgitant flow through appropriate management strategies, which may include surgical intervention such as valve replacement in severe cases such as this.

Surgical intervention is crucial in managing *Streptococcus sanguinis* endocarditis, mainly when severe valvular dysfunction and regurgitation occur. The evidence in the medical literature supports the importance of timely surgical intervention in cases like this, aiming to address the underlying infection, restore valve function, and prevent further complications [9]. Published studies have consistently shown that early surgical intervention, such as valve replacement in this case, is associated with improved outcomes in patients with infective endocarditis [9]. The decision to proceed with the surgery is based on several factors, including the severity of valvular dysfunction, complications such as abscess formation or embolization, and the patient’s overall clinical condition. In this case, severe regurgitation from *Streptococcus sanguinis* endocarditis necessitated surgical intervention. The regurgitant flow significantly burdens the heart, leading to progressive cardiac remodeling and compromised cardiac function. Surgical replacement of the affected valve aims to restore normal valve function and eliminate the regurgitant flow, thus relieving the strain on the heart and improving overall cardiac performance.

In this case, the first valve replacement addressed the initial infection caused by *Streptococcus sanguinis* endocarditis. However, small mobile vegetations on the atrial aspect of the anterior leaflet indicated persistent infection and inadequate clearance of the bacteria. These were likely a result of incomplete eradication of the infectious process. The persistence of vegetation after the initial valve replacement underscores the challenges in managing *Streptococcus sanguinis* endocarditis. This highlights the tenacity of *Streptococcus sanguinis* as a pathogen and its ability to resist treatment measures, potentially leading to recurrent infections. The need for a second procedure underscores the severity and debilitating consequences of *Streptococcus sanguinis* endocarditis, as it entails additional surgical risks and prolongs the patient’s recovery and rehabilitation process.

The patient, in this case, was treated with a monotherapy of intravenous ceftriaxone for six weeks to eliminate the endocardium infection. Studies have shown that cephalosporins alone can be effective [10-13], and such an approach was taken for six weeks. Intravenous ceftriaxone is also highly effective in treating infective endocarditis and is recommended as a first-line agent by the Infectious Diseases Society of America (IDSA) guidelines for treating infective endocarditis. The usual duration of treatment for infective endocarditis caused by *S. sanguinis* is 4-6 weeks, which may be adjusted based on the patient’s clinical response. It is important to note that the choice of antibiotic therapy was based on the susceptibility profile of the organism and the patient’s clinical condition. The mortality rate for infective endocarditis caused by *S. sanguinis* has been reported to be low, with the majority of deaths occurring in patients with complicated diseases or delayed diagnosis and treatment. In terms of outcomes, the prognosis for infective endocarditis caused by *S. sanguinis* is generally favorable if treated promptly and appropriately. However, developing complications may result in a more complicated clinical course and worse outcomes.

## Conclusions

In conclusion, the presented case of *Streptococcus sanguinis* endocarditis in a 26-year-old female highlights this condition’s rare and debilitating nature. The need for two mitral valve replacements due to persistent infection and vegetation development after the first procedure further exemplifies the challenges and complexities of *Streptococcus sanguinis* endocarditis. This case serves as a reminder of the potential complications and poor outcomes that can arise from this rare infection. It emphasizes the importance of timely and aggressive surgical intervention in managing severe valvular dysfunction and controlling the infection.
